# Comparison of Inflammatory Effects in THP-1 Monocytes and Macrophages after Exposure to Metal Ions

**DOI:** 10.3390/ma13051150

**Published:** 2020-03-05

**Authors:** Henrike Loeffler, Anika Jonitz-Heincke, Kirsten Peters, Brigitte Mueller-Hilke, Tomas Fiedler, Rainer Bader, Annett Klinder

**Affiliations:** 1 Biomechanics and Implant Technology Research Laboratory, Department of Orthopedics, Rostock University Medical Centre, Doberaner Strasse 142, 18057 Rostock, Germany; henrike.loeffler@uni-rostock.de (H.L.); anika.jonitz-heincke@med.uni-rostock.de (A.J.-H.); rainer.bader@med.uni-rostock.de (R.B.); 2 Department of Cell Biology, Rostock University Medical Center, Schillingallee 69, 18057 Rostock, Germany; kirsten.peters@med.uni-rostock.de; 3 Institute for Immunology, Rostock University Medical Center, Schillingallee 70, 18057 Rostock, Germany; brigitte.mueller-hilke@med.uni-rostock.de; 4 Institute for Medical Microbiology, Virology and Hygiene, Rostock University Medical Center, Schillingallee 70, 18057 Rostock, Germany; tomas.fiedler@med.uni-rostock.de

**Keywords:** aseptic loosening, corrosion, metal ions, monocytes, macrophages, inflammation

## Abstract

Monocytes and macrophages are the first barrier of the innate immune system, which interact with abrasion and corrosion products, leading to the release of proinflammatory mediators and free reactive molecules. The aim of this study was to understand inflammation-relevant changes in monocytes and macrophages after exposure to corrosion products. To do this, the THP-1 cell line was used to analyze the effects of metal ions simultaneously in monocytes and differentiated macrophages. Cells were stimulated with several concentrations of metal salts (CoCl_2_, NiCl_2_, CrCl_3 _× 6H_2_O) to analyze viability, gene expression, protein release and ROS production. Untreated cells served as negative controls. While exposure to Cr(3+) did not influence cell viability in both cell types, the highest concentration (500 µM) of Co(2+) and Ni(2+) showed cytotoxic effects mirrored by significantly reduced metabolism, cell number and a concomitant increase of ROS. The release of IL-1β, IL-8, MCP-1 and M-CSF proteins was mainly affected in macrophages after metal ion exposure (100 µM), indicating a higher impact on pro-inflammatory activity. Our results prove that monocytes and macrophages react very sensitively to corrosion products. High concentrations of bivalent ions lead to cell death, while lower concentrations trigger the release of inflammatory mediators, mainly in macrophages.

## 1. Introduction

Degenerative joint diseases contribute to the decrease in quality of life during aging. Due to increased aging in the general population, therapeutic measures, including total joint replacement, progressively gain importance in tackling these diseases [1]. One major problem associated with total joint replacement is the necessity of revisions caused by the septic and aseptic loosening of the implant. According to Herberts et al., aseptic loosening accounts for more than 70% of knee prosthesis failures and 44% of hip prosthesis failures [2]. In order to improve the long-term outcome after total joint replacement, orthopedic research targets elevating the implant success rates and understanding the reasons for revision.

One approach is to improve implant materials by understanding the mechanisms at the implant surface responsible for inflammation and loosening. Commonly used materials in prosthesis manufacturing are metal alloys, because of their high mechanic stability and good biological compatibility. Main materials are stainless steel (consisting of iron, chromium, nickel), cobalt-chromium–molybdenum and titanium [3]. Due to abrasion and corrosion processes, wear particles and metal ions occur in periprosthetic tissue [4]. 

These wear products interact with the defense barrier of innate immunity that is mainly driven by monocytes and macrophages. Phagocytosis of wear particles by macrophages is considered the beginning of these reactions, finally resulting in endoprosthesis failure [4,5]. Secretion of proinflammatory cytokines, osteomodulating mediators as well as reactive oxygen and nitrogen species initiates and maintains the inflammation processes around the implant [4].

Initial immune response to particle exposition is mainly accompanied by the production of proinflammatory TNF-α, chemokine IL-8, and cytokines IL-1β and IL-6 [6]. The release of monocyte chemotactic protein 1 (MCP-1) triggers the recruitment of more and more monocytes, thus further promoting inflammation processes [4,7,8]. Besides the invading monocytes, the release of various differentiation factors like receptor activator of NF-κB ligand (RANKL) and monocyte colony-stimulating factor (M-CSF) also triggers the maturation of local macrophages and their differentiation into osteoclasts [9]. Thus, in vivo, there is always a combination of mature macrophages and freshly invading monocytes that drive the inflammation. Often, only one cell type was investigated, or reactivity was attributed equally to both of them. However, considering that after phagocytosis of metallic wear particles by mature macrophages, these macrophages release metal ions from their lysosomes into their microenvironment [10], they might be better equipped to withstand the effects of those ions. Therefore, we hypothesized that macrophages might react differently to metal ions compared to monocytes. 

Therefore, our study aimed to understand inflammation-relevant changes in monocytes and macrophages after exposure to corrosion products. The use of the THP-1 cell line, which can be cultured as monocytes but also differentiated into macrophages, allowed us to analyze the effects of metal ions in parallel with monocytes and macrophages from the same origin. In the in vitro investigation, viability assays as well as gene and protein expression analyses and the quantification of reactive oxygen species (ROS) after metal salt exposure were carried out. 

## 2. Materials and Methods 

### 2.1. Preparation of Metal Salt Solutions

The following metal salts were purchased from Sigma-Aldrich (Sigma-Aldrich Chemie GmbH, Munich, Germany): Cobalt(II) chloride (purum p.a., anhydrous, purity ≥ 98.0% (KT)), Nickel(II) chloride (anhydrous, powder, purity 99.99% trace metals basis) and Chromium(III) chloride hexahydrate (purum p.a., purity ≥ 98.0% (RT)) and stock solutions of a concentration of 100 mM were produced as described previously [11]. For cell culture experiments, the stock solutions were diluted with cell culture media to various concentrations. 

### 2.2. Cell Culture

THP-1 monocytes were cultivated in Roswell Park Memorial Institute (RPMI) 1640 medium supplemented with 20% fetal calf serum (FCS; Pan Biotech GmbH, Aidenbach, Germany), 2% L-Alanyl-L-Glutamin (Biochrom GmbH, Berlin, Germany), 1% amphotericin b and 1% penicillin/streptomycin (both: Sigma-Aldrich, Munich, Germany) at 37 °C and 5% CO_2_. 

For each experiment, cells were seeded into two cell culture plates. While cells of one plate remained in suspension, cells of another plate were differentiated for 24 h using 100 ng/mL Phorbol-12-myristat-13-acetat (PMA; Sigma-Aldrich, Munich, Germany), so monocytes and macrophages could be examined simultaneously. Cells were stimulated with several concentrations of metal salts and viability, and gene expression and protein biosynthesis analyses as well as ROS assay were carried out. Untreated cells served as negative controls.

The effects of metal salts were tested after 48 h incubation, since longer incubation periods were not feasible. As found by us (data not shown) and also reported by Lund et al. [12], THP-1 derived macrophages de-differentiate, detach from the surface of the cell culture dish and show a round monocyte-like morphology after more than 48 h without PMA. However, the simultaneous presence of PMA in THP-1 macrophage culture was shown to interfere with the effects of nickel ions [13]. We therefore decided to differentiate for 24 h with PMA, to then remove PMA from the cell culture and to limit the incubation time with metal ions to 48 h. 

### 2.3. Cellular Activity

The viability of THP-1 monocytes and macrophages after exposure to metal ions was determined by the metabolic activity assay WST-1 (Roche, Penzberg, Germany) and CyQUANT NF Cell Proliferation Assay (Invitrogen (Thermo Fisher Scientific), Waltham, MA, USA).

A total of 10,000 cells per well were seeded into black 96-well cell culture plates (Thermo Fisher Scientific Inc., Waltham, MA, USA). Cells were treated with 10, 50, 100 and 500 µM of metal ions for 48 h. For the determination of cell activity, ion solution was removed and cells were incubated with a defined volume of WST-1/medium reagent (ratio 1:10) at 37 °C and 5% CO_2_ for 30 min. Afterwards, supernatants of the respective culture medium were transferred into 96-well cell culture plates (ThermoFisher Scientific, Waltham, MA, USA) to measure the absorption at 450 nm (reference wave length: 630 nm) in a microplate reader (Tecan Reader Infinite^®^ 200 Pro, Tecan Trading AG, Maennedorf, Switzerland)

CyQUANT cell proliferation assay was performed to determine the absolute cell number according to the manufacturer’s recommendations. Cells were covered with 100 µL 1× Dye Binding Solution (consisting of 1:500 Dye Reagent and 1× HBSS) and incubated for 60 min, protected from light. Fluorescence intensity was measured at 530 nm (excitation wavelength: 485 nm) using the Tecan-Reader Infinite^®^ 200 Pro. In order to relate the fluorescence signal to an actual cell number, a cell number calibration curve was prepared with previously defined cell numbers in duplicate. 

Cellular activity was calculated by dividing WST-1 results by the respective cell number. 

### 2.4. Analysis of Gene Expression

The following experimental setup was used to determine gene as well as protein expression and formation of reactive oxygen species: 60,000 cells per well of a 24-well cell culture plate were treated with 100 µM of metal salts for 48 h. Untreated cells served as negative control for metal salt exposure. Supernatants were collected and stored at −20 °C.

RNA was isolated using the peqGOLD Total line RNA Kit and the related manufacturer’s protocol (VWR International GmbH, Hanover, Germany). RNA was eluted into a fresh sterile tube using RNase-free water and RNA concentration was measured using the Tecan Reader Infinite^®^ 200 Pro microplate reader and NanoQuantTM Plate (Tecan Trading AG, Maennedorf, Switzerland) with RNase-free water as blank. Afterwards, RNA was transcribed into amplifiable cDNA using the High Capacity cDNA Reverse Transcription Kit (Applied Biosytems, Foster City, CA, USA) according to manufacturer’s recommendations. A total of 50 ng RNA was attained by transferring appropriate amounts of RNA-containing sample into PCR tubes and adding RNase free water to reach a volume of 10 µL. A total of 10 µL of master mix was added and PCR was carried out using following RT-PCR protocol: 10 min at 25 °C, 120 min at 37 °C, 15 s at 85 °C in a thermocycler (Analytik Jena, Jena, Germany). Afterwards, samples were diluted in additional 20 µL RNase free water and stored at −20 °C.

Relative quantification of target cDNA levels was done by semi-quantitative realtime PCR (qTower 2.0, Analytik Jena AG, Jena/Germany) using innuMIX qPCR MasterMix SyGreen (Analytik Jena AG, Jena, Germany) and the primers (Sigma-Aldrich, Darmstadt, Germany), as listed in Table 1. 

A master mix was prepared for each gene, containing 0.5 µL of forward and reverse primer, 3 µL Aqua dest. and 5 µL of SyGreen qPCR MasterMix. A total of 1 µL of template cDNA of each sample was pipetted onto the bottom of a 96-well PCR plate in duplicates and filled up with 9 µL of master mix. RNase-free water served as a negative control. The plate was sealed with adhesive foil and placed in the qTower 2.0. qPCR was performed under the following conditions: 2 min at 95 °C and 40 cycles of 95 °C (5 s) and 65 °C (25 s). A cycle threshold (Ct) of 30 was set as the limit of interpretation. The relative expression of each mRNA compared with the housekeeping gene HPRT was calculated by the equation ∆Ct = Ct_target _− Ct_HPRT_. The relative amount of target mRNA in the unstimulated cells and treated cells was expressed as 2^(−∆∆Ct)^, where ∆∆Ct = ∆Ct_treated_ − ∆Ct_control_. 

### 2.5. Quantification of Cytokine Release in Cell Culture Supernatants

The protein contents of interleukin 1β (IL-1β), unterleukin 8 (IL-8), monocyte chemotactic protein 1 (MCP-1) and macrophage colony-stimulating factor (M-CSF) were quantified in cell culture supernatants using corresponding ELISA Ready-SET-Go! Kits (ThermoFisher Scientific, Waltham, MA, USA) according to the manufacturer’s recommendations. Absorbance was measured at 450 nm (reference wave length: 570 nm). Sample concentrations were calculated using a standard curve and set in ratio to total protein concentrations, quantified by the Qubit Protein Assay Kit and Qubit 1.0 (both: Invitrogen, Waltham, MA, USA). 

### 2.6. ROS Assay

To detect the presence of total free-reactive oxygen species, the OxiSelect™ In vitro ROS/RNS Assay (Cell Biolabs, Inc., San Diego, CA, USA) was used. Firstly, medium supernatants of exposed monocytes and macrophages were centrifuged. Afterwards, 50 µL of standard or sample were transferred into black 96-well cell culture plates (Thermo Fisher Scientific Inc., Waltham, MA, USA) and incubated with a catalyst that sped up the oxidative reaction. Next dichlorodihydrofluorescin was added to the samples. Finally, samples were fluorometrically measured against the standard curve to determine the content of ROS. 

### 2.7. Statistical Analyses

Statistical and graphic data interpretation was performed using GraphPad Prism 7.02 (GraphPad Software Inc., San Diego/USA). 

Cellular viability assay results are shown as box plots. Boxes depict interquartile ranges, horizontal lines within boxes depict medians and whiskers depict maximum and minimum values. Viability assay data were interpreted using repeated measures two-way ANOVA followed by Bonferroni multiple comparison tests.

ROS assay results are shown as mean ± SD, including the single datapoints related to the untreated control values set as 100%. Statistical analyses were performed using the reactive oxygen species amounts divided by control values. Statistical analysis was performed using two-way ANOVA and Bonferroni’s multiple comparison test as the post hoc test.

Gene expression results are shown as mean ± SD, including the single datapoints as percentage of 2^(−∆∆Ct)^ with untreated controls set as 100%. Statistical analysis was performed using two-way ANOVA with the ∆Ct values and Bonferroni’s multiple comparison test as the post hoc test. 

Protein expression values are shown as mean ± SD, including the single datapoints normalized to total protein. For these data, there is no depiction related to the untreated controls as, for some of the determined proteins, no protein release was detected in the untreated controls. Statistical analyses were performed using the protein concentration values normalized to total protein. Two-way ANOVA and Bonferroni’s multiple comparisons test were performed.

## 3. Results

### 3.1. Effects of Metal Ions on Cell Number and Cellular Activity in THP-1 Monocytes and Macrophages

**Figure 1:**
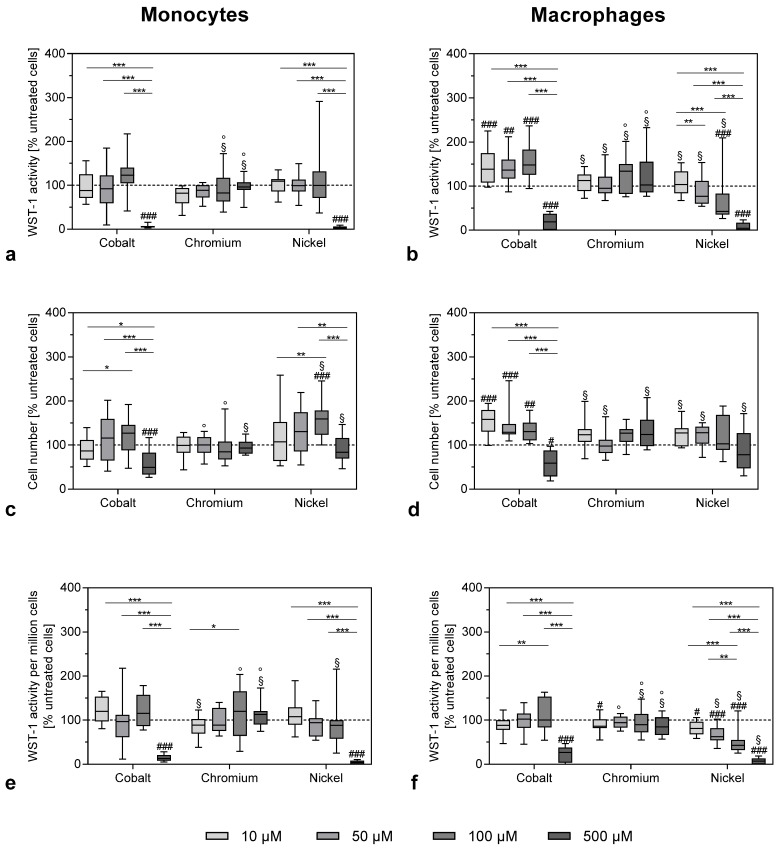
Cell viability of THP-1 monocytes (**a**–**c**) and macrophages (**d**–**f**) after exposure to metal salts. WST-1 assay (**a**,**b**) and CyQUANT NF Cell Proliferation Assay (**c**,**d**) were carried out after 48 h of stimulation with different concentrations of cobalt, chromium and nickel ions. Figure 1e,f show the WST-1 activity per 1,000,000 cells. All values were normalized to untreated cells and shown as a percentage, with negative control set as 100% (dotted line). Data (n = 12) are shown as box plots with minimum, 25th percentile, median, 75th percentile and maximum values. Significant differences were calculated by repeated measures two-way ANOVA and Bonferroni’s multiple comparisons test as post hoc test from the original values (non-normalized). Significantly different between different concentrations: * p < 0.05, ** p < 0.01, *** p < 0.001; significantly different from untreated control: # p < 0.05, ## p < 0.01, ### p < 0.001; significantly different from cobalt at the same concentration: §; significantly different from nickel at the same concentration: °.

After exposure to the highest concentration of 500 µM, the metabolic cell activity of monocytes was reduced by cobalt and nickel ions as measured by WST-1 conversion assay (Figure 1a) or metabolic activity normalized to the cell number (Figure 1e). 

The total cell number was less affected by the treatment with cobalt and nickel ions (Figure 1c). However, it has to be taken into consideration that, due to the nature of the CyQuant assay, which quantifies cell-number-based fluorescence labelling of DNA content, dead or damaged cells were also counted. Exposure to chromium ions at the used concentrations had no effect on cell activity or cell number in monocytes (Figure 1a–c). Similar results were observed in the THP-1 macrophages (Figure 1b,d,f) with Ni(2+) exposure, showing a clear concentration-dependent reduction (Figure 1f). When comparing the effects between monocytes and macrophages, there were no significant differences for the higher concentrations. However, lower concentrations of cobalt ions led to an increase in WST-1 activity and cell number in macrophages (monocytes vs. macrophages: p = 0.0089 and p = 0.0049 for WST-1 activity at 10 and 50 µM cobalt ions, respectively, as well as p < 0.0001 for cell number at 10 µM cobalt ions).

**Figure 2:**
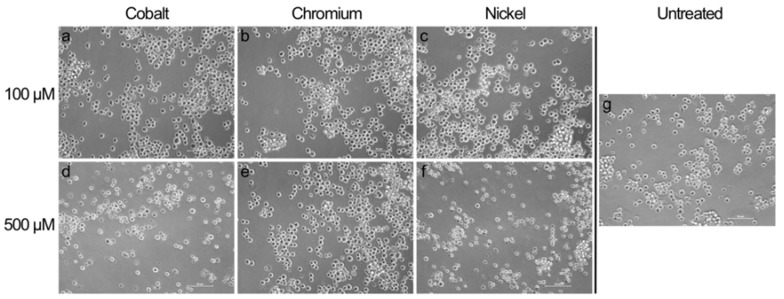
Light microscopic pictures of THP-1 monocytes exposed to metal salts and untreated cells. Monocytes were treated with 100 µM (**a**–**c**) and 500 µM (**d**–**f**) of cobalt, chromium and nickel ions. Untreated cells are depicted in Figure 2g. Pictures were taken after 48 h of exposure in 20× magnification. Scale bar = 50 µm.

**Figure 3:**
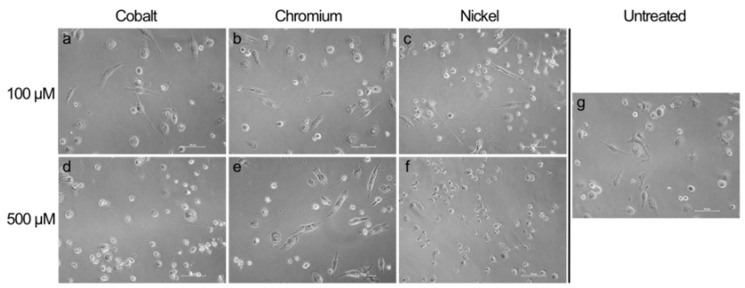
Light microscopic pictures of THP-1 macrophages exposed to metal salts and untreated cells. Macrophages were treated with 100 µM (**a**–**c**) and 500 µM (**e**–**g**) of cobalt, chromium and nickel ions). Untreated cells are depicted in Figure 3g. Pictures were taken after 48 h of exposure in 20× magnification. Scale bar = 50 µm.

The result that concentrations of 500 µM of cobalt and nickel ions were quite toxic for monocytes as well as macrophages was confirmed by the morphological changes observed by light microscopy. Monocytes as well as macrophages showed a more irregular shape after 500 µM cobalt and nickel ion exposure (Figure 2 and Figure 3).

Macrophage attachment seemed less strong after cobalt and nickel treatment with a higher concentration and, instead of the spread morphology that was observed in untreated controls and for lower concentrations, here, macrophages showed a rounded cell shape similar to that of monocytes (Figure 3e,g). Chromium ion exposure had no visible effect compared to untreated controls.

### 3.2. Effects of Metal ions on ROS Production in THP-1 Monocytes and Macrophages

**Figure 4:**
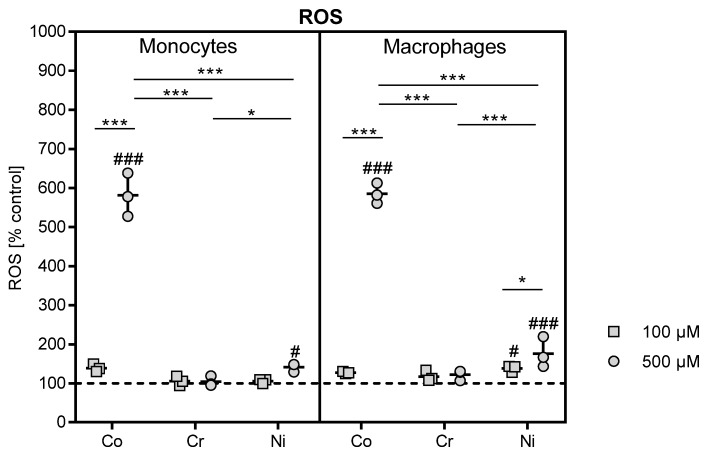
Reactive oxygen species (ROS) quantification in cell culture supernatants. THP-1 monocytes and macrophages were treated with different concentrations of cobalt, chromium and nickel ions over 48 h. Untreated cells served as negative control. ROS formation was determined in cell culture supernatants using OxiSelect™ In vitro ROS/RNS Assay. Data are shown as percentage in relation to control cells (100%, dotted line) as single datapoints with mean ± SD (n = 3). Significances between groups were calculated with Two-way ANOVA and Bonferroni’s multiple comparisons test as a post hoc test. Significantly different between concentrations or different metal salt treatments at the same concentration: * p < 0.05, ** p < 0.01, *** p < 0.001; significantly different from untreated control: # p < 0.05, ## p < 0.01, ### p < 0.001.

The determination of reactive oxygen species in 100 and 500 µM cobalt-, chromium- and nickel ion-treated THP-1 monocytes and macrophages revealed a massive increase in ROS formation in reaction to exposure to the higher cobalt ion concentration for monocytes and macrophages in comparison to control cells (both: p < 0.0001) as well as to 500 µM chromium- and nickel-stimulated cells (p < 0.0001), respectively (Figure 4). While reaction to nickel ion exposure in THP-1 monocytes only showed an association in the 500 µM stimulated sample (p = 0.0384), macrophages showed a stronger reaction to nickel ion treatment. Exposure to 100 and 500 µM nickel increased in ROS release into the supernatant compared to untreated control (p = 0.0231 and p < 0.0001, respectively) in THP-1 macrophages, and the effect was more pronounced for 500 compared to 100 µM nickel ion exposure (p = 0.0203). Incubation with 500 µM nickel ions also led to a higher release than observed in 500 µM chromium-stimulated macrophages (p = 0.0004).

### 3.3. Influence of Metal Ions on Gene Expression and Protein Release of Inflammatory Mediators in THP-1 Monocytes and Macrophages

As exposure to cobalt and nickel at the concentration of 500 µM was shown to be toxic for monocytes and macrophages, the experiments regarding the effects of metal ion on gene and protein were only carried out with the highest subtoxic concentration of 100 µM.

#### 3.3.1. Gene Expression and Protein Release of the Proinflammatory Cytokine IL-1β

**Figure 5:**
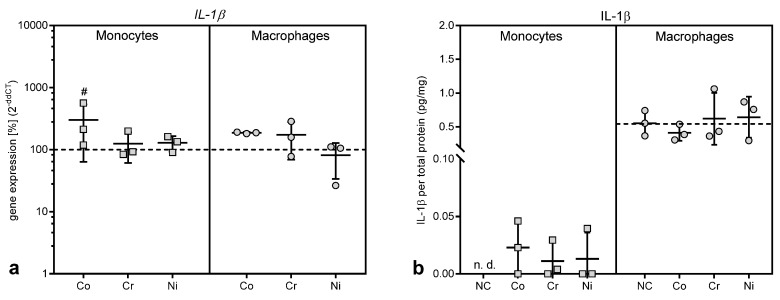
Gene expression and protein release of interleukin (IL-) 1β following exposure to metal salts. Gene expression (**a**) and protein release into cell culture supernatants (**b**) was determined for THP-1 monocytes and macrophages after 48 h of treatment with the concentration of 100 µM of cobalt, chromium and nickel salts. Untreated cells served as negative controls. Data (n = 3) are depicted as single datapoints with mean ± SD. Gene expression data are shown as percentage of untreated cells (2^(−∆∆Ct)^, 100%, dotted line), while protein release data represent values of the specific protein amount normalized to total protein content in cell culture supernatants. Significances between groups were calculated with two-way ANOVA with Bonferroni post hoc test using ∆CT values for gene expression and specific protein amount normalized to total protein content for protein release in cell culture supernatants. Significantly different from untreated control: # p < 0.05, ## p < 0.01, ### p < 0.001. Abbreviations: n.d. = not detectable; NC = negative control (untreated cells).

While the gene expression of *IL-1β* was upregulated after exposure to 100 µM cobalt ions (Figure 5a), this effect only reached significance in THP-1 monocytes (p = 0.0304 compared to untreated control, Bonferroni post hoc test). There were no significant differences regarding *IL-1β* gene expression between monocytes and macrophages. The increase in *IL-1β* gene expression in THP-1 monocytes was mirrored by increased protein release into the cell culture medium (Figure 5b). Indeed, only after exposure to metal ions IL-1β protein release was detected, while untreated monocytes did not release IL-1β (p = 0.0429 for variable “treatment” in two-way ANOVA). 

Interestingly, THP-1 macrophages showed IL-1β protein release already at baseline, i.e., in the untreated macrophages (Figure 5b), and the protein production was not influenced by the treatment with metal ions. The release of IL-1β from THP-1 macrophages was considerably higher than from monocytes (monocytes vs. macrophages: p = 0.0415, p = 0.0159 and p = 0.0109 for untreated cells, as well as treatment with chromium and nickel ions, respectively). 

#### 3.3.2. Gene Expression and Protein Release of Chemokines

**Figure 6:**
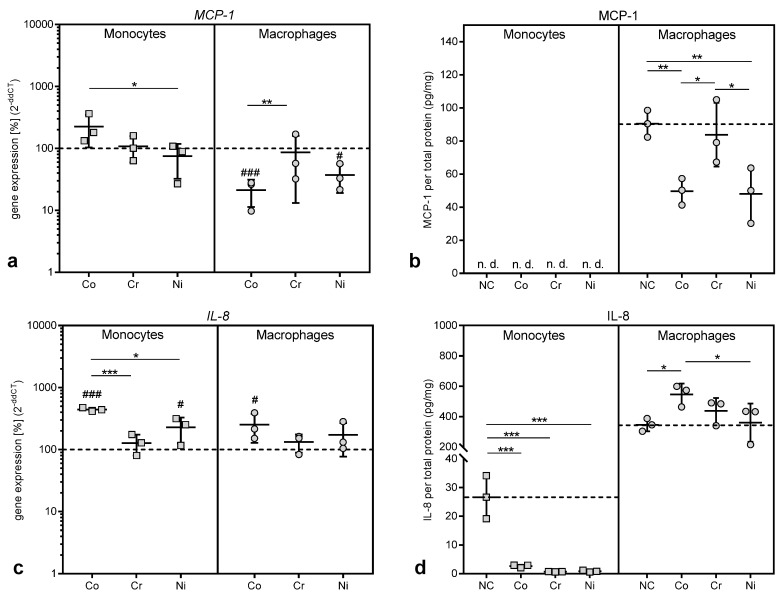
Gene expression and protein release of monocyte chemotactic protein (MCP-) 1 (**a**,**b**) and Interleukin (IL-) 8 (**c**,**d**) following exposure to metal salts. Gene expression (**a**,**c**) and protein release into cell culture supernatants (**b**,**d**) were determined for THP-1 monocytes and macrophages after 48 h of treatment with the concentration of 100 µM of cobalt, chromium and nickel salts. Untreated cells served as negative controls. Data (n = 3) are depicted as single datapoints with mean ± SD. Gene expression data are shown as percentage of untreated cells (2^(−∆∆Ct)^, 100%, dotted line) while protein release data represent values of the specific protein amount normalized to total protein content in cell culture supernatants. Significances between groups were calculated with two-way ANOVA and Bonferroni’s multiple comparison test as post hoc test using ∆CT values for gene expression and specific protein amount normalized to total protein content for protein release in cell culture supernatants. Significantly different between stimulation groups: * p < 0.05, ** p < 0.01, *** p < 0.001; significantly different from untreated control: # p < 0.05, ## p < 0.01, ### p < 0.001. Abbreviations: n.d. = not detectable; NC = negative control (untreated cells).

MCP-1 is a chemokine which is responsible for the recruitment of monocytes, but also of natural killer cells and T-lymphocytes, to the location of inflammation [4,8,14,15]. While gene expression of *MCP-1 *was slightly, but non-significantly, increased in THP-1 monocytes after exposure to cobalt ions (Figure 6a), no protein release into the cell culture supernatant for MCP-1 was detected in monocytes (Figure 6b). Contrary to the monocytes, treatment with cobalt and nickel ions led to a significant downregulation of *MCP-1* gene expression in THP-1 macrophages (p = 0.0005 and p = 0.0142 for Co and Ni vs. untreated control, Figure 6a). This decrease was also observed regarding the release of MCP-1 protein from macrophages (p = 0.0049 and p = 0.0036 for Co and Ni vs. untreated control, Figure 6b). Exposure to chromium ions did not show any effects.

IL-8 is another chemokine initially involved in the recruitment of neutrophilic granulocytes and macrophages [4,7,8]. Exposure to cobalt and nickel ions resulted in an upregulation of *IL-8* gene expression in THP-1 monocytes and macrophages compared to untreated cells (p = 0.0001 and p = 0.0238 for Co and Ni in monocytes; p = 0.0179 for Co in macrophages). Chromium ions did not affect *IL-8* gene expression (Figure 6c). While the increased gene expression resulted in an increased protein release for IL-8 in macrophages, protein release was significantly reduced in THP-1 monocytes after exposure to all three metal ions compared to untreated controls, despite increased levels of gene expression (p = 0.0004, p = 0.0002 and p = 0.0002 for Co, Cr and Ni vs. untreated control in THP-1 monocytes, Figure 6d).

#### 3.3.3. Gene Expression and Protein Release of Mediators of Macrophage Differentiation

**Figure 7:**
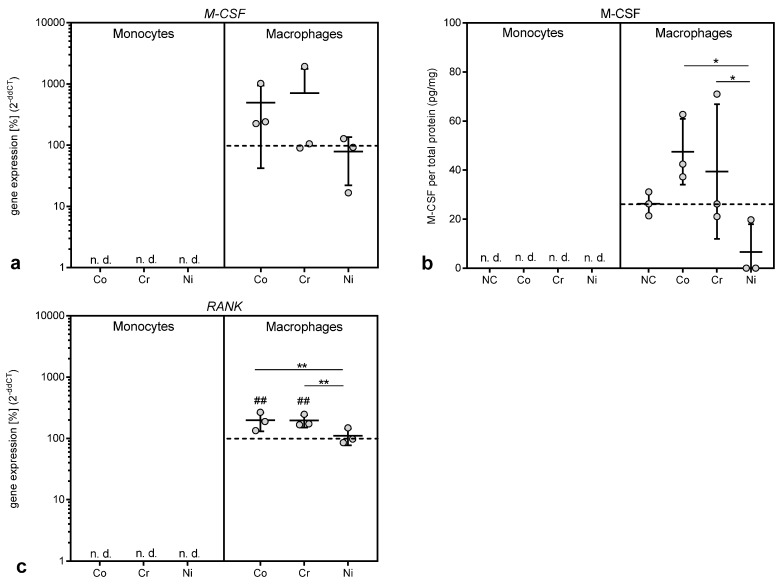
Gene expression and protein release of macrophage colony-stimulating factor (M-CSF) (**a**,**b**) and receptor activator of NF-κB (RANK) (**c**) following exposure to metal salts. Gene expression (**a**,**c**) and protein release into cell culture supernatants (**b**) was determined for THP-1 monocytes and macrophages after 48 h of treatment with the concentration of 100 µM of cobalt, chromium and nickel salts. Untreated cells served as negative controls. Data (n = 3) are depicted as single datapoints with mean ± SD. Gene expression data are shown as percentage of untreated cells (2^(−∆∆Ct)^, 100%, dotted line) while protein release data represent values of the specific protein amount normalized to total protein content in cell culture supernatants. Significances between groups were calculated with two-way ANOVA and Bonferroni’s multiple comparison test as post hoc test using ∆CT values for gene expression and specific protein amount normalized to total protein content for protein release in cell culture supernatants. Significantly different between stimulation groups: * p < 0.05, ** p < 0.01, *** p < 0.001; significantly different from untreated control: # p < 0.05, ## p < 0.01, ### p < 0.001. Abbreviations: n.d. = not detectable; NC = negative control (untreated cells).

M-CSF and RANK are both proteins involved in the differentiation and maturation of macrophages, the latter especially in the differentiation into osteoclasts [5,14]. Gene expression of both mediators was only detectable in THP-1 macrophages but not in monocytes (Figure 7a,c). Exposure to cobalt and chromium ions increased gene expression of *M-CSF* and *RANK*, with the association reaching significance for *RANK* (p = 0.0030 and p = 0.0024 for Co and Cr vs. untreated control, respectively). The upregulation of *M-CSF* in THP-1 macrophages was mirrored by an increased release of the protein after cobalt and chromium treatment (Figure 7b). It is notable that chromium ions, which did not show any effects in the other assays presented here, affected both mediators.

## 4. Discussion

In our study, the effects on THP-1 monocytes and macrophages from metal ions at concentrations ranging from 10 to 500 µM were investigated. The evidence that these concentrations are indeed similar to concentrations found in periprosthetic tissue was discussed in detail in a previous publication [11]. The analysis of cell number and cellular activity revealed the impact of metal ions on metabolic activity by showing a significant decrease in activity that was mainly prominent in 500 µM cobalt- and nickel ion-treated cells. Similar results for nickel ions were reported by Chana et al. [13]. The loss of metabolic activity was accompanied by a massive release of reactive oxygen species. The mechanisms of concentration-dependent cytotoxicity for cobalt and nickel ions are based on their ability to interfere with DNA replication and DNA repair after cell membrane penetration, and these changes finally initiate necrosis [16]. These events are partially mediated by reactive oxygen species. It was suggested that metal ions potentiate electron exchange reactions and induce radical formation [17]. Reactive oxygen species are signal molecules produced and broken down in the cells under physiological conditions. Increased production of reactive oxygen species is originally applied by cells of the innate immune system to fight pathogens and initiate pathogen destruction [18]. However, if the production of reactive oxygen species exceeds the elimination capacity of cells, ROS accumulate and induce oxidative cell stress. Cobalt and nickel ions were able to induce ROS production and might therefore cause oxidation of cellular proteins, lipids and DNA, finally leading to cell damage and cell death [19]. Thus, the phagocytosis of particles or elevated cell stress induced by exposure to corrosion product, i.e., high concentrations of cobalt and nickel ions, results in overshooting ROS production that can lead to the observed decrease in cell activity, or even to cell destruction, as indicated by the light microscopy images [20,21]. 

In contrast, chromium ions did not cause a significant decrease in cell activity. Investigations by Ferko et al. [22] and Kwon et al. [16] further support the assumption of cobalt and nickel ions having a stronger impact on monocytes and macrophages in the applied concentrations. Cell death studies by Huk et al. [23] proved that chromium ion toxicity needed much higher concentrations than in cobalt stimulation to manifest. Bivalent cobalt ions, for example, can penetrate cell membranes passively using different ion channels [24]. Trivalent chromium ions, as used in this study, are not known to possess mechanisms like this. That chromium(III) ions have no known processes of uptake via the cell membrane might also impact on ROS production, as in the chromium-stimulated samples no increase in ROS was observed. In solution, they form aggregates in the cell culture medium. However, very high concentrations of chromium ions that exceed the investigated range may be able to induce hypoxia in the cell and cause a decrease in viability [22,25]. However, since chromium (VI) ions exhibit up to 1000-fold higher toxicity than chromium(III) ions, our results may not adequately reflect the in vivo effects of chromium [26]. In the body, chromium ions may also be present in other forms, such as chromate [CrO_4_]_2_− or dichromate, during aseptic loosening [27]. Chromate is the predominant form of chromium 6+ in solutions and is able to cross cell membranes through nonspecific anion carriers [17,26]. This may be the reason for the observed in vivo toxicity of chromium components. It is also possible that Cr(3+) in the investigated concentrations might be involved in aseptic loosening via other mechanisms, as, indeed, chromium ions were able to increase the gene expression of mediators of macrophage differentiation *M-CSF* and *RANK* in our study. This finding was rather surprising, as Cr(3+) at the investigated concentration showed no cellular or metabolic effects in this nor in a previous study in osteoblasts [11]. While M-CSF is known to drive differentiation of macrophages into a M2 phenotype [28], and might thus rather ameliorate the inflammation, RANK as the membrane bound receptor for the RANK ligand is directly involved in the initiation and persistence of osteolysis [4,5,7,29,30]. The induction of RANK might thus link chromium ions to the osteolysis processes observed in aseptic loosening.

Apart from chromium ions, cobalt ions were also able to upregulate *RANK* expression in THP-1 macrophages. Indeed, cobalt ions had the most pronounced effects in all the performed experiments. While the highest concentration of 500 µM resulted in a loss of cell number and WST-1 activity, in both THP-1 monocytes and macrophages, the lower concentrations of 10, 50 and 100 µM cobalt ions specifically increased proliferation and cellular activity in THP-1 macrophages. This effect might also be due to the low-level oxidative stress and the production of ROS as, for example, the stimulation of macrophage proliferation by ceramide 1-phosphate was mediated through the generation of ROS [31]. It is possible that the stimulation of proliferation and metabolic activity is a means by which macrophages adapt to the changed microenvironment and fulfill their function as “cleaners” in the body [32,33]. However, this seems to be a finely balanced process, as the higher ROS concentrations observed after nickel treatment of THP-1 macrophages were already cytotoxic.

The induction of oxidative stress in macrophages is furthermore considered a main cause of cytokine release. A recent review by Hallab and Jacobs [7] summarized the danger signal pathway that finally leads to the release of mature IL-1β, IL-18, IL-33, and other cytokines and chemokines as follows. The “inflammasome” pathway senses “danger-associated molecular patterns” and induces danger signaling through mechanisms such as lysosomal destabilization. The cascade of nicotinamide adenine dinucleotide phosphate (NADPH) oxidase caused by the lysosomal destabilization and an associated increase in reactive oxygen species in turn activate the intracellular multi-protein “inflammasome” complex composed of NALP3 (NACHT-, LRR-, and pyrin domain-containing protein 3) in association with apoptosis-associated speck-like protein containing a CARD domain (ASC). This further activates Caspase-1, which, in this case, does not act as an apoptosis stimulus but rather converts cytokines such as IL-1β and IL-18 from their inactive into their active form. This mechanism could account for the observation that IL-1β release was only detected in monocytes after treatment with metal salts as the activation of IL-1β secretion requires a “second signal” [34]. However, THP-1 macrophages already showed a high basic IL-1β release in the untreated cells, which was not influenced by metal ion treatment. It cannot be ruled out that the artificial situation in cell culture, e.g., the attachment to the cell culture dish or the differentiation with Phorbol-12-myristat-13-acetat, provided enough stimulus for IL-1β secretion. Danger signaling might not be the only mechanisms involved in IL-1β secretion, as it was shown that HIFα, which is stabilized by cobalt ions [24], is involved in IL-1β biosynthesis [35]

Interestingly, MCP-1, which was upregulated in debris-induced inflammation [7], was shown to be decreased in gene expression as well as protein biosynthesis after treatment with cobalt and nickel ions in THP-1 macrophages, and was not released from THP-1 monocytes at all despite increased gene expression. A similar effect was observed in THP-1 monocytes for IL-8, which showed a significant elevation in gene expression level, while the protein release was significantly decreased compared to untreated controls. In THP-1 macrophages, however, we found the expected increase in *IL-8* mRNA as well as IL-8 protein after incubation with cobalt ions [4,8,11,36]. To our knowledge, a mechanism for the suppression of IL-8 release in monocytes by metal ions has not been described and we can only speculate that it represents a self-limiting mechanism in inflammation by reducing the further recruitment of neutrophilic granulocytes and monocytes once metallic debris is broken down into ions by macrophages. We assume that the reduction in MCP-1 and IL-8 protein in some of the supernatants, which contradicted the increased gene expression results, was due to reduced protein synthesis and release, e.g., by influencing the mRNA stability, which has been described as one mechanism to fine-tune chemokine availability [37]. However, other fine-tuning mechanisms may also influence the availability of chemokines in the supernatants. It was reported that both chemokines can bind to atypical chemokine receptors [38]. For example, the binding of a chemokine to D6, which is an atypical receptor for MCP-1, leads to the internalization of the receptor-ligand complex followed by the rapid degradation of the ligand [38,39].

We can conclude from this study that metal ions induced different effects in monocytes and macrophages, which were especially apparent for the concentration of inflammatory mediators in the supernatants. We cannot rule out that the use of a cell line influenced our results and further experiments in primary human monocytes and macrophages have to be carried out to confirm the results.

## Figures and Tables

**Table 1:** Primer sequences for qPCR.

Primer	Sequences (5′-3′)
Hypoxanthine-guanine phosphoribosyl transferase (HPRT)	*for*: CCCTGGCGTCGTGATTAGTG *rev*: TCGAGCAAGACGTTCAGTCC
Interleukin 1β (IL-1β)	*for:* TACTCACTTAAAGCCCGCCT *rev*: ATGTGGGAGCGAATGACAGA
Interleukin 8 (IL-8)	*for:* TCTGTGTGAAGGTGCAGTTTTG *rev:* ATTTCTGTGTTGGCGCAGTG
Monocyte chemotactic protein 1 (MCP-1)	*for: *CCGAGAGGCTGAGACTAACC *rev:* GGCATTGATTGCATCTGGCTG
Macrophage colony-stimulating factor (M-CSF)	*for:* TCCAGCCAAGATGTGGTGAC *rev: *AGTTCCCTCAGAGTCCTCCC
